# Trace Metal Impurities Effects on the Formation of [^64^Cu]Cu-diacetyl-bis(*N*^4^-methylthiosemicarbazone) ([^64^Cu]Cu-ATSM)

**DOI:** 10.3390/ph17010010

**Published:** 2023-12-21

**Authors:** Mitsuhiro Shinada, Hisashi Suzuki, Masayuki Hanyu, Chika Igarashi, Hiroki Matsumoto, Masashi Takahashi, Fukiko Hihara, Tomoko Tachibana, Chizuru Sogawa, Ming-Rong Zhang, Tatsuya Higashi, Hidemitsu Sato, Hiroaki Kurihara, Yukie Yoshii, Yoshihiro Doi

**Affiliations:** 1 Faculty of Science, Toho University, Funabashi 274-8510, Japan; takahasi@chem.sci.toho-u.ac.jp (M.T.); 7321201t@st.toho-u.jp (T.T.); yoshihiro.doi@sci.toho-u.ac.jp (Y.D.); 2 Institute for Quantum Medical Science, National Institutes for Quantum Science and Technology, Chiba 263-8555, Japan; suzuki.hisashi@qst.go.jp (H.S.); hanyu.masayuki@qst.go.jp (M.H.); cigaras2023@gmail.com (C.I.); matsumoto.hiroki2@qst.go.jp (H.M.); fukiko.hihara@gmail.com (F.H.); sogawa.chizuru@qst.go.jp (C.S.); zhang.ming-rong@qst.go.jp (M.-R.Z.); higashi.tatsuya@qst.go.jp (T.H.); 3 Kanagawa Cancer Center, Kanagawa 241-8515, Japan; satohidemitsu@hotmail.com (H.S.); h-kurihara@kcch.jp (H.K.)

**Keywords:** trace metal impurities, [^64^Cu]Cu-ATSM, quality management

## Abstract

[^64^Cu]Cu-diacetyl-bis(*N*^4^-methylthiosemicarbazone) ([^64^Cu]Cu-ATSM) is a radioactive hypoxia-targeting therapeutic agent being investigated in clinical trials for malignant brain tumors. For the quality management of [^64^Cu]Cu-ATSM, understanding trace metal impurities’ effects on the chelate formation of ^64^Cu and ATSM is important. In this study, we conducted coordination chemistry studies on metal–ATSM complexes. First, the effects of nonradioactive metal ions (Cu^2+^, Ni^2+^, Zn^2+^, and Fe^2+^) on the formation of [^64^Cu]Cu-ATSM were evaluated. When the amount of Cu^2+^ or Ni^2+^ added was 1.2 mol or 288 mol, equivalent to ATSM, the labeling yield of [^64^Cu]Cu-ATSM fell below 90%. Little effect was observed even when excess amounts of Zn^2+^ or Fe^2+^ were added to the ATSM. Second, these metals were reacted with ATSM, and chelate formation was measured using ultraviolet–visible (UV-Vis) absorption spectra. UV-Vis spectra showed a rapid formation of Cu^2+^ and the ATSM complex upon mixing. The rate of chelate formation by Ni^2+^ and ATSM was lower than that by Cu-ATSM. Zn^2+^ and Fe^2+^ showed much slower reactions with the ATSM than Ni^2+^. Trace amounts of Ni^2+^, Zn^2+^, and Fe^2+^ showed little effect on [^64^Cu]Cu-ATSM’ quality, while the concentration of impurity Cu^2+^ must be controlled. These results can provide process management tools for radiopharmaceuticals.

## 1. Introduction

Hypoxia has been implicated in the poor prognosis of malignant tumors, such as recurrent brain tumors [1,2,3,4]. Extensive hypoxic areas are associated with a poor prognosis in patients, as malignant factors are promoted in hypoxic cancer cells [2,3,4,5]. Therefore, new tumor therapeutics targeting hypoxia have extensively been investigated to improve patient outcomes. We developed a promising radioactive hypoxia-targeting theranostic agent, [^64^Cu]Cu-diacetyl-bis(*N*^4^-methylthiosemicarbazone) ([^64^Cu]Cu-ATSM). Clinical studies on patients with recurrent brain tumors are underway.

**Figure 1:**
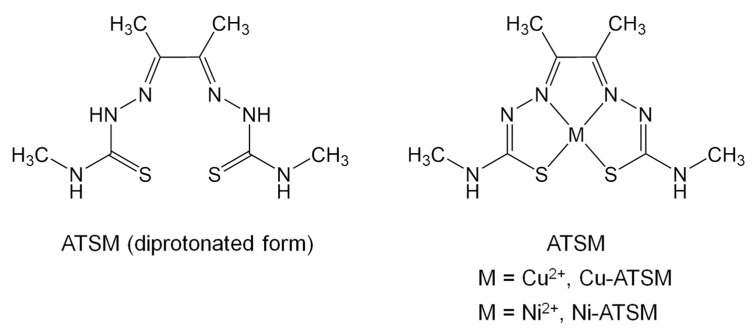
ATSM (free ligand form) and its Cu^2+^ and Ni^2+^ complexes.

Cu-ATSM (Figure 1) is labeled with several copper isotopes, such as ^60^Cu, ^61^Cu, ^62^Cu, and ^64^Cu, for positron emission tomography (PET) imaging under hypoxia [6,7,8,9,10,11,12,13,14]. Cu-ATSM rapidly permeates cells, is reduced, and is trapped within cells under hypoxia [15,16,17,18,19]. In hypoxic cells, the levels of the biological reductant NAD(P)H and the activity of NAD(P)H-dependent reductive enzymes are upregulated, and highly reduced conditions lead to Cu-ATSM retention in the cells [16,20,21,22]. To develop hypoxia-targeting radiotherapeutics, ^64^Cu was chosen to label ATSM. ^64^Cu emits β+ for PET imaging (0.655 MeV, 17.8%), β^-^ (0.574 MeV, 40%), and particularly, Auger electrons bearing a high potential for killing cancer cells [21].

In our previous study [22], using a xenograft mouse model of glioblastoma, multiple doses of [^64^Cu]Cu-ATSM significantly prolonged survival, but no toxicity was reported [22]. We stabilized the therapeutic dose of [^64^Cu]Cu-ATSM by adding sodium L-ascorbate to our investigational drug formulation [23]. We also evaluated trace amounts of chemical impurities derived from the degradation of ATSM in our formulation of [^64^Cu]Cu-ATSM [24]. We identified chemical impurities derived from the degradation of ATSM by using liquid chromatography with tandem mass spectrometry (LC-MS/MS). We also assessed their chemical hazards using quantitative structure–activity relationship (QSAR) applications and concluded that the potential risk posed by chemical impurities contained in the therapeutic dose of [^64^Cu]Cu-ATSM is negligible.

Cu-64 was obtained by the ^64^Ni(p,n)^64^Cu reaction using a cyclotron. After the dissolution of the ^64^Ni target with hydrochloric acid, no carrier added (n.c.a.) ^64^Cu was obtained by chemical separation using an anionic exchange resin or cationic exchange resin [25,26,27,28]. Although the ^64^Ni target was carefully purified before irradiation, minimal amounts (10^0^–10^2^ ppb) of impurities such as Cu, Ni, Fe, and Zn remained in the irradiated sample solution. Most metal ions are present as divalent ions in solution and may react competitively with ATSM to form [^64^Cu]Cu-ATSM chelates, thereby inhibiting [^64^Cu]Cu-ATSM’s formation.

Our formulation of the [^64^Cu]Cu-ATSM investigational drug contains 2.5 µg/mL of ATSM and up to 1.5 GBq/mL of ^64^Cu [23]. The molar ratio of ATSM to n.c.a. ^64^Cu in the therapeutic formulation was approximately 60. Therefore, sub-ppm-level contamination of metal ions may negatively affect the radiochemical yield of [^64^Cu]Cu-ATSM. As of date, the effect of trace metal impurities on chelate formation by ^64^Cu has been reported for some ligands by Ferreira et al. (DOTA-DBCB) [29], Boswell et al. (H_2_CB-TE2A) [30], and Zeng et al. (EdF-DOTA) [31]; however, that for ATSM remains unclear. Therefore, control of trace metal impurities would be critical for the quality management of ^64^Cu radiopharmaceutical agents. To obtain quantitative insights into the chelate formulations of ATSM and metal ions, we conducted studies on the coordination chemistry of metal–ATSM complexes in this study. First, we investigated the effect of non-radioactive metal ions on the formation of [^64^Cu]Cu-ATSM. Copper, nickel, zinc, and iron were chosen for this experiment because they are possible contaminants in the target system of the cyclotron [32]. Next, these metals were made to react with ATSM, and the chelate formation rate was measured using ultraviolet–visible (UV-Vis) absorption spectra. These results can provide useful process management tools for this promising radiopharmaceutical for the treatment of recurrent brain tumors in future clinical studies.

## 2. Results

### 2.1. Experiment 1: Effect of Trace Metal Impurities on the Formation of [^64^Cu]Cu-ATSM Complex

**Figure 2:**
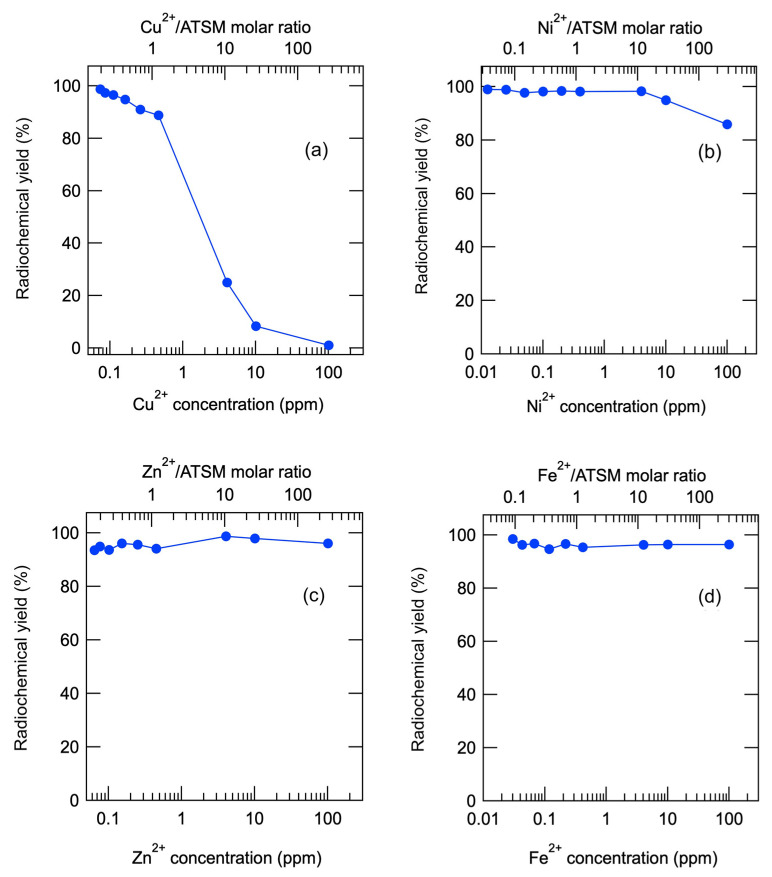
Effect of radiochemical yield of [^64^Cu]Cu-ATSM on coexisting metal ions. (**a**): Cu^2+^, (**b**): Ni^2+^ (**c**): Zn^2+^, (**d**): Fe^2+^.

We investigated the radiochemical yield of [^64^Cu]Cu-ATSM when metal solutions (Cu^2+^, Ni^2+^, Zn^2+^, and Fe^2+^) at nine different concentrations (0.0125, 0.025, 0.05, 0.1, 0.2, 0.4, 4, 10, and 100 ppm in 40.5 μL of the reaction mixture) were added during the synthesis. The concentration range was set at approximately 100 times the amount considered to be contaminated based on the literature [26,32]. The results are summarized in Figure 2 and Table 1 and Table 2. Figure 2 shows the plots of the radiochemical yield of [^64^Cu]Cu-ATSM against the concentration of metal ions in the solutions. The top axis shows the molar ratio of the metal ions to ATSM. Table 1 and Table 2 show the concentration of metal ions, radiochemical yield, and molar ratio of M^2+^ to ATSM (i.e., M^2+^/ATSM). As shown in Figure 2a, Cu^2+^ inhibited the formation of the [^64^Cu]Cu-ATSM complex at lower concentrations compared to other metal ions. When 1.2 mol equivalent of Cu^2+^ or more was added to ATSM, the radiochemical yield of [^64^Cu]Cu-ATSM was reduced to lower than 90%, which is regarded as a quality standard for radiopharmaceuticals.

Figure 2b shows the concentration-dependent effects of Ni^2+^ on [^64^Cu]Cu-ATSM complex formation. The radiochemical yield of [^64^Cu]Cu-ATSM fell below 90% upon adding 288 equivalent Ni^2+^ ions to ATSM. Thus, the effect of Ni^2+^ on [^64^Cu]Cu-ATSM formation was 240 times weaker than that of Cu^2+^. Little effects of Zn^2+^ and Fe^2+^ were observed, even with the addition of 248 mol or 290 mol equivalents of ATSM (Figure 2c and 2d, respectively, and Table 2).

### 2.2. Experiment 2: Chelate Formation of ATSM with Metal Ions

**Figure 3:**
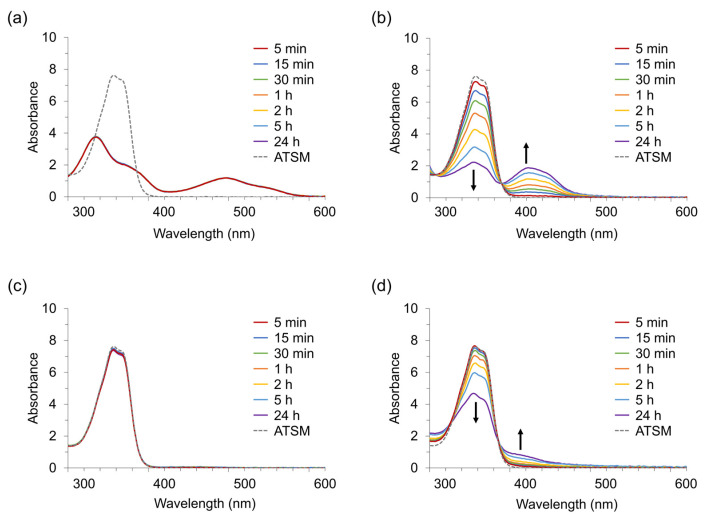
Variation in UV-Vis spectra of DMSO solution containing 300 µM MCl_2_ and 300 µM-ATSM at room temperature with time. A 300 µM (90 nmol) MCl_2_ concentration equals an M^2+^ concentration of Cu^2+^ (19.1 ppm), Ni^2+^ (17.6 ppm), Zn^2+^(19.6 ppm), and Fe^2+^ (16.8 ppm). (**a**): Cu-ATSM, (**b**): Ni-ATSM, (**c**): Zn-ATSM, (**d**): Fe-ATSM.

In Experiment 2, we examined chelate formation when metal ions (Cu^2+^, Ni^2+^, Zn^2+^, and Fe^2+^) were added to the ATSM with UV-Vis spectra over time. Figure 3 shows spectral changes in the ATSM after adding transition metal ions. Cu^2+^ chelated ATSM within 5 min. Ni^2+^ also formed chelates with ATSM; however, the reaction rate was slower than that with Cu^2+^. In the case of Fe^2+^, the ATSM peak at 337 nm gradually decreased, and a weak shoulder appeared at around 400 nm, indicating a slower reaction rate of Fe^2+^ than that of Ni^2+^. The spectrum of Zn^2+^ remained unchanged after 24 h, indicating a prolonged reaction rate.

**Figure 4:**
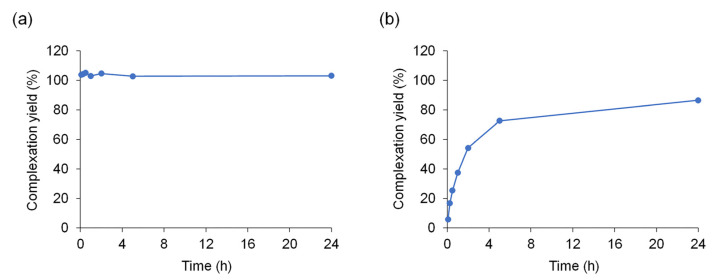
Time-dependent increase in M-ATSM concentration during the reaction of 300 μM MCl_2_ with 300 μM ATSM in DMSO at room temperature. (**a**) Cu-ATSM, (**b**) Ni-ATSM.

The reactions for Cu^2+^ and Ni^2+^ were studied semi-quantitatively; the concentrations of Cu- and Ni-ATSM were estimated using the molar extinction coefficients, ε, which were determined at 7680 (Cu-ATSM at 477 nm) and 14,300 cm M^–1^ (Ni-ATSM at 402 nm) in this study (Figure S1). Although the ε values in DMSO have not been reported, the values in DMF (Cu-ATSM) [33] and pyridine (Ni-ATSM) [34] are similar to our findings. Figure 4 shows the time-dependent increase in the M-ATSM concentration; the vertical axis represents the concentration of M-ATSM relative to the complete complex concentration. The concentration of Cu-ATSM reached 100% immediately after 5 min, suggesting the completion of the chelate formation. In contrast, the complexation yield of Ni^2+^ with ATSM gradually increased from 5.8% at 5 min to over 50% at 2 h, indicating that the formation of Ni-ATSM is slower and weaker than that of Cu-ATSM.

### 2.3. Experiment 3: Chelate Formation of Zn-ATSM in the Presence of Brønsted Base

**Figure 5:**
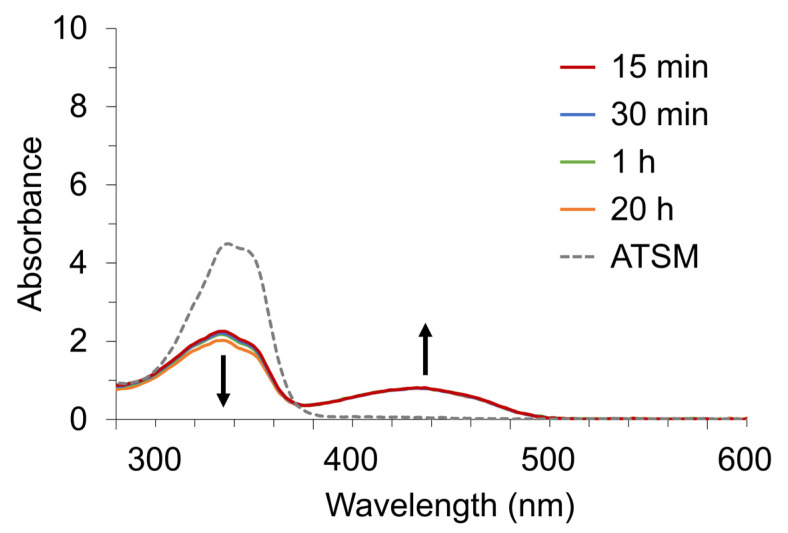
Time-dependent change in the UV-Vis spectra of DMSO solution containing 300 µM ZnCl_2_, 300 µM ATSM, and 600 µM sodium methoxide at room temperature.

In Experiment 2, the UV-Vis spectrum of ATSM showed that only Zn^2+^ did not react under this formulation condition. To understand the underlying reason, we hypothesized that deprotonation of ATSM would be necessary for coordination with Zn^2+^, and, therefore, we checked whether the use of a Brønsted base changes the reaction rate of Zn-ATSM. Figure 5 shows the changes in the UV-Vis spectrum of ATSM after mixing with ZnCl_2_ and sodium methoxide as a Brønsted base. In contrast to the Zn-ATSM spectrum shown in Figure 3c, Zn-ATSM began to form 15 min after the initiation of the reaction. The ATSM peak slightly decreased for 20 h, but that of Zn-ATSM did not change from 15 min to 20 h.

### 2.4. Experiment 4: Differences in the Formation Constants of Cu-ATSM and Ni-ATSM

**Figure 6:**
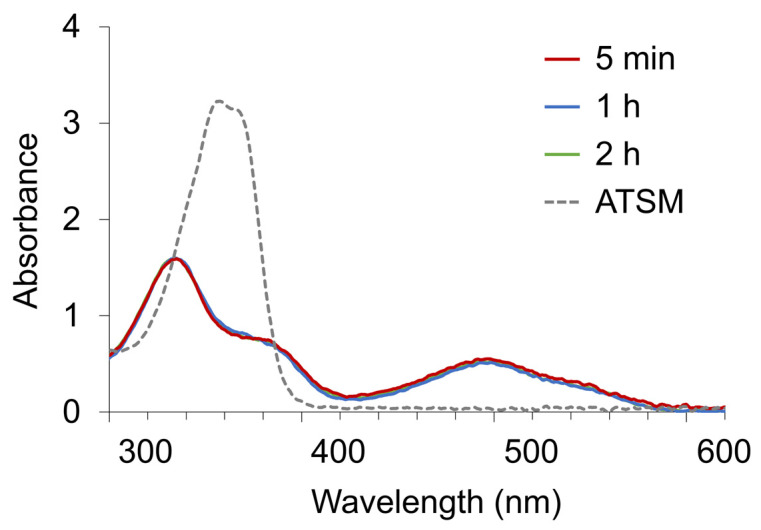
Time-dependent changes in the UV-Vis spectra of ATSM after simultaneously mixing with Cu^2+^ and Ni^2+^.

To confirm the difference of the formation constants between Cu^2+^ and Ni^2+^ with ATSM, 150 μM CuCl_2_, 150 μM NiCl_2_, and 150 μM, ATSM were mixed in a total volume of 600 µL, and the UV-Vis spectra change was obtained (Figure 6). Compared to Figure 3a,b, the Cu-ATSM spectrum was observed within 5 min of the start of the reaction, and there was no significant change in the spectrum after 2 h, suggesting that the formation constant of Cu-ATSM was much larger than that of Ni-ATSM. The results showed that upon mixing Cu^2+^ and Ni^2+^, the chelate formation rates of ATSM and Cu^2+^ were much higher than those of Ni^2+^, and the formation of Cu-ATSM was thermodynamically predominant.

## 3. Discussion

[^64^Cu]Cu-ATSM is a promising therapeutic radiopharmaceutical for clinical trials. The ^64^Cu used in [^64^Cu]Cu-ATSM was prepared using a cyclotron and contained trace metal impurities, mainly Cu^2+^, Ni^2+^, Zn^2+^, and Fe^2+^, derived from cyclotron target systems and production lines. Therefore, to obtain insights into the effects of trace metal impurities on the chelate formation of ^64^Cu and ATSM ligands, we conducted coordination chemistry studies on metal–ATSM complexes.

Experiment 1 indicates that trace amounts of Cu^2+^ and Ni^2+^ affect the radiochemical yield of [^64^Cu]Cu-ATSM. In contrast, Fe^2+^ and Zn^2+^ did not affect radiochemical yield, even when the concentration of these metals reached up to 100 ppm. This result is consistent with those of Experiment 2 (vide infra). Defined as a specification for the investigational drug formulation of [^64^Cu]Cu-ATSM, the radiochemical yield of the chelate should be not less than 90%. Considering this criterion, the maximum metal ion concentrations for [^64^Cu]Cu-ATSM formation were 0.26 ppm (260 ppb) for Cu^2+^, 10 ppm for Ni^2+^, and 100 ppm for Fe^2+^ and Zn^2+^. These values were defined as the highest experimental concentrations at which radiochemical yield could be maintained above 90% in the presence of the impurities.

The metal impurities in the irradiated ^64^Ni target were estimated using previously reported values [26,32,35,36]. The concentration of metal impurities can differ among laboratories because it depends on the cyclotron irradiation, mainly on the target and subsequent separation process. The ^64^Cu source was produced at our institute, QST, and we referred to the values of Ohya et al. [32]. The reported concentrations in 10 mL solutions were Cu: 36 ± 22, Ni: 12 ± 27, Fe: 64 ± 48, and Zn: 157 ± 42 ppb. Assuming that the impurities came from only 10 mL of the solution recovered from the target and that all impurities were in the final product, the estimated concentrations of the metal impurities in our products were well below the recommended levels mentioned above, indicating that the metal impurities from the target did not affect the radiochemical yields in our formulation. In this study, the concentration of Cu^2+^ impurities in the reaction mixture derived from the target system was estimated to be 60.8 ppb in the reaction mixture. This value was approximately 20% of the allowed value (260 ppb). In the production process of ^64^Cu, the ^64^Ni fraction is usually recovered to regenerate as the target; as a result, the purity of the ^64^Ni target increases, and control of the copper impurities in the target is essential. According to the values of metal impurities reported by other institutes, the copper content reaches the permissible value in one case; thus, in such a case, the removal of Cu^2+^ from the ^64^Ni target using a chelate resin, for instance, is required [37,38].

The chelate formation reaction was examined qualitatively from a kinetic point of view, with contrasting results obtained for Cu^2+^ and Ni^2+^. Cu^2+^ was made to quantitatively react with ATSM within 5 min, whereas Ni^2+^ showed a slow reaction. The formation of Ni-ATSM complexes was almost complete after 24 h, but the amount of formed Ni-ATSM was only 86% of that of the theoretical amount from the quantitative reaction. This observation suggests that the system was in chemical equilibrium, and Ni^2+^ did not react quantitatively with ATSM. Thus, the reaction of metal ions with the ATSM is more favorable with Cu^2+^ than with Ni^2+^. The low inhibition of Ni^2+^ and ATSM can be explained by both thermodynamic and kinetic consequences.

The reaction of Zn^2+^ with ATSM showed little spectral change, suggesting a slow reaction rate for Zn^2+^. One reason for the slow reaction may be that deprotonation of ATSM is required to coordinate with Zn^2+^. The reaction proceeded more rapidly when we added two equivalents of sodium methoxide, a strong Brønsted base (Figure 5). This finding suggests that a kinetic factor is involved, whereby Zn^2+^ does not inhibit the formation of the Cu-ATSM complex. Therefore, deprotonating agents may inhibit the ions involved in [^64^Cu]Cu-ATSM formation, and the concentration of deprotonating agents in the [^64^Cu]Cu-ATSM formulation must be strictly controlled.

The kinetic experiments indicated that the reaction rates are different between Cu^2+^ and Ni^2+^. This phenomenon is frequently observed in the formation of metal complexes in aqueous solutions. The exchange reaction rate of the solvent molecule dominated most of the formation reaction rates, and no ligand could react faster than the solvent molecule. A significant difference in the solvent exchange reaction in aqueous solutions was observed between Cu^2+^ and Ni^2+^; the exchange rate constant, *k*_ex_, of Cu^2+^ was 10^5^ times larger than that of Ni^2+^ in water [39]. We expected a similar relationship with DMSO concentration.

Under the conditions of Experiment 1, the maximally allowed Ni^2+^ concentration of 10 ppm corresponded to 170 μM, and the molar ratio Ni^2+^/ATSM was 29. This indicated that Ni^2+^ did not inhibit [^64^Cu]Cu-ATSM formation up to 29 equivalents to ATSM. This observation suggests the low chelate formation ability of Ni^2+^, although the coexisting glycine, added as an auxiliary complexing agent to maintain Cu^2+^ in the solution, can weaken the coordination of ATSM to some extent through the competition of ATSM with glycine. This coexisting glycine also competed with [^64^Cu]Cu-ATSM formation because the molar ratio of total Cu, the sum of cold Cu and ^64^Cu, to ATSM was only 0.68 at 91% radiochemical yield.

The effect of metal ions on the formation of [^44^Sc]Sc-DOTATATE (Figure S2) has been reported [40]. Two nmol/mL of Fe^2+/3+^ and Zn^2+^ and 10 nmol/mL of Cu^2+^ significantly reduced the labeling yield, while Ca^2+^ and Al^3+^ did not affect it up to 2 μmol/mL. The formation of [^44^Sc]Sc-DOTATATE was more sensitive to metal ion contamination, in contrast to our [^64^Cu]Cu-ATSM. This high inhibition of DOTATATE results from the fact that the DO3A moiety of DOTATATE can coordinate to a wide range of M^2+^ and M^3+^ ions with high formation constants, reducing the selectivity of DOTATATE.

A limitation of this study is that the formation constant of the ATSM complex was not determined. However, these values can be referred to for the same coordination atom. The formation constant, *K*_f_, for Ni^2+^ and Cu^2+^ complexes of 1,10-diaza-4,7-dithiadecane (Figure S3) has been reported; the log *K*_f_ value is 7.41 for Ni^2+^ and 10.70 for Cu^2+^ [41]. These values indicated that the ligand formed a more stable (approximately 1000 times) complex with Cu^2+^ than with Ni^2+^. The values of ATSM can be larger than 1,10-dizaza-4,7-dithiodecane because of the rigid framework of the ligand, and the same stability relationship holds for ATSM. The Irving–Williams series can interpret this stability relationship. The Irving–Williams series shows that the order of stability of the divalent transition metal complex of a given ligand is Mn^2+^ < Fe^2+^ < Co^2+^ < Ni^2+^ < Cu^2+^ > Zn^2+^ [42]. The hard and soft acid and base theory [43] can also explain the large formation constant of Cu-ATSM. Fe^2+^, Ni^2+^, Cu^2+^, and Zn^2+^ are Lewis acids that are intermediates between hard and soft acids, and Cu^2+^ has the highest electronegativity and polarizability among these ions. This indicates that Cu^2+^ is the softest acid in the series and has the highest affinity for soft Lewis bases containing sulfur atoms. Thus, ATSM has the highest affinity for Cu^2+^, and the inhibition of the formation of [^64^Cu]Cu-ATSM by Ni^2+^, Fe^2+^, and Zn^2+^ can be negligible in future manufacturing practices.

## 4. Materials and Methods

### 4.1. Reagents and Materials

All reagents and solvents from commercial sources (Fujifilm Wako Pure Chemical, Osaka, Japan) were of high quality and were used without further purification. ATSM and Cu-ATSM were purchased from ABX Advanced Biochemical Laboratories (Radeberg, Germany). Japan Pharmacopoeia grade distilled water (Otsuka Pharmaceutical Factory, Tokyo, Japan) was used for preparation of aqueous solutions. Spectrochemical-analysis-grade dimethyl sulfoxide (DMSO) was purchased from Fujifilm Wako Pure Chemical Co., Ltd., Osaka, Japan. The Ni^2+^ complex of ATSM was prepared following a previously reported method [44].

### 4.2. Determination of Radiochemical Yield

The radiochemical yield of [^64^Cu]Cu-ATSM was determined by radio-TLC. One microliter of the sample solution was spotted onto an HPTLC silica gel 60 F_254_ glass plate (Merck Millipore, Burlington, MA, USA) and developed using methanol. Radioactivity on the TLC plates was measured using a radio-TLC system (Raytest PET miniGITA Star; Elysia s.a., Liège, Belgium).

### 4.3. Experiment 1: Effect of Trace Metal Impurities on the Formation of [^64^Cu]Cu-ATSM Complex

The experiments were performed on a scale of 1/400 of the clinical formulation, and the concentrations of the reagents, including ^64^Cu and ATSM, were kept constant.

Cyclotron-produced ^64^CuCl_2_ (29.25 MBq; 3.20 pmol) was dissolved in 20 μL of 0.125 M aqueous glycine solution, and 0.5 μL of 0.5 mM ATSM in DMSO was added and mixed well. Then, 20 μL of each metal aqueous solution (Cu^2+^, Ni^2+^, Zn^2+^, and Fe^2+^) at nine different concentrations (0.0125, 0.025, 0.05, 0.1, 0.2, 0.4, 4, 10, and 100 ppm in 40.5 μL of the reaction mixture) were added. The mixed solutions were incubated at room temperature for 10 min. The radiochemical yield of [^64^Cu]Cu-ATSM was determined by radio-TLC. The effect of adding these metal solutions was evaluated by plotting the radiochemical yield against the concentration of metal ions as the sum of the added metal ions and metal impurities derived from the target system.

### 4.4. Experiment 2: Chelate Formation of ATSM with Metal Ions

Briefly, 300 μM of metal solutions (Cu^2+^, Ni^2+^, Zn^2+^, and Fe^2+^) were prepared by dissolving their chlorides into DMSO, and 300 µL of each solution was mixed with 300 µL of 300 μM ATSM in DMSO. UV-Vis spectra were measured at 5, 15, 30, 1, 2, 5, and 24 h after mixing. Spectra were measured using a NanoDrop One Microdrop UV-Vis Spectrometer (ND-ONE-W; Thermo Fisher Scientific, Waltham, MA, USA) with 1 μL of each mixed solution. The molar absorption coefficient (ε) of Cu-ATSM and Ni-ATSM was determined on 100 μM DMSO solutions of Cu-ATSM and Ni-ATSM. The concentrations of the Cu-ATSM and Ni-ATSM in each reaction mixture were analyzed based on the obtained ε values.

### 4.5. Experiment 3: Chelate Formation of Zn-ATSM in the Presence of Brønsted Base

The spectrum of the reaction between ATSM and Zn^2+^ remained almost unchanged for 24 h in Experiment 2. One possible reason for the slow reaction is that deprotonation of ATSM is required to coordinate with Zn^2+^. Therefore, we confirmed whether the reaction rate of Zn-ATSM is changed by using the Brønsted base. A total of 600 µL of DMSO solution containing 200 µL of a ZnCl_2_ (300 µM), 200 µL of ATSM (300 µM), and 200 µL of sodium methoxide (600 µM) was stirred at room temperature. The UV-Vis spectra were measured at 15 min, 30 min, 1 h, and 20 h after mixing. The spectra were measured using the same method as in Experiment 2.

### 4.6. Experiment 4: Differences in the Formation Constants of M-ATSM in the Reaction of ATSM with Cu^2+^ and Ni^2+^ Mixtures

Specifically, DMSO solutions of 150 µM CuCl_2_ (200 µL) and 150 µM NiCl_2_ (200 µL) were mixed, and 200 µL solution of ATSM in DMSO (150 µM) was added at room temperature. Changes in UV-Vis spectra were measured at 5 min, 1 h, and 2 h after mixing. The spectra were measured using the same method as in Experiment 2.

## 5. Conclusions

This study showed that 1.2 mol Cu^2+^ or 288 mol Ni^2+^, equivalent to ATSM, decreased the labeling yield of [^64^Cu]Cu-ATSM below 90%. Zn^2+^ and Fe^2+^ had little effect on the labeling of [^64^Cu]Cu-ATSM, even with the addition of 248 or 290 mol equivalents of ATSM. Based on these data, we conclude that trace amounts of Ni^2+^, Zn^2+^, and Fe^2+^ have little effect on the quality of [^64^Cu]Cu-ATSM; however, the concentration of Cu^2+^ must be controlled. These results can provide process management tools for radiopharmaceuticals.

## Figures and Tables

**Table 1:** Effect of coexisting transition metal ions (Cu^2+^ and Ni^2+^) on radiochemical yield of [^64^Cu]Cu-ATSM. The contents of metal ions in the reaction mixture, shown as the unit of ppm and mol in parenthesis, are indicated as the sum of added metal ions and metal impurities derived from the target system.

Cu^2+^			Ni^2+^		
Metal Ion in ppm (mol)	Radiochemical Yield (%)	Ratio ^(1)^	Metal Ion in ppm (mol)	Radiochemical Yield (%)	Ratio
0.073 (1.9 × 10^−8^)	98.8	0.19	0.0125 (3.4 × 10^−9^)	99.0	0.036
0.086 (2.2 × 10^−8^)	97.4	0.22	0.025 (6.9 × 10^−9^)	98.8	0.072
0.11 (2.9 × 10^−8^)	96.6	0.28	0.05 (1.4 × 10^−8^)	97.7	0.14
0.16 (4.1 × 10^−8^)	94.8	0.41	0.1 (2.8 × 10^−8^)	98.2	0.29
0.26 (6.7 × 10^−8^)	91.0	0.66	0.2 (5.5 × 10^−8^)	98.4	0.58
0.46 (1.2 × 10^−7^)	88.8	1.2	0.4 (1.1 × 10^−7^)	98.2	1.2
4.1 (1.0 × 10^−6^)	25.0	10	4 (1.1 × 10^−6^)	98.3	12
10 (2.6 × 10^−6^)	8.3	26	10 (2.8 × 10^−6^)	94.9	29
100 (2.6 × 10^−5^)	1.0	255	100 (2.8 × 10^−5^)	85.9	288

^(1)^ Metal ion (mol)/ATSM (mol).

**Table 2:** Effect of coexisting transition metal ions (Zn^2+^ and Fe^2+^) on radiochemical yield of [^64^Cu]Cu-ATSM. The contents of metal ions in the reaction mixture, shown as the unit of ppm and mol in parenthesis, are indicated as the sum of added metal ions and metal impurities derived from the target system.

Zn^2+^			Fe^2+^		
Metal Ion in ppm (mol)	Radiochemical Yield (%)	Ratio	Metal Ion in ppm (mol)	Radiochemical Yield (%)	Ratio
0.064 (1.6 × 10^−8^)	93.5	0.16	0.030 (8.8 × 10^−9^)	98.5	0.088
0.077 (1.9 × 10^−8^)	94.9	0.19	0.043 (1.2 × 10^−8^)	96.4	0.12
0.10 (2.5 × 10^−8^)	93.7	0.25	0.068 (2.0 × 10^−8^)	96.7	0.20
0.15 (3.8 × 10^−8^)	96.1	0.38	0.12 (3.4 × 10^−8^)	94.6	0.34
0.25 (6.3 × 10^−8^)	95.7	0.62	0.22 (6.3 × 10^−8^)	96.6	0.63
0.45 (1.1 × 10^−7^)	94.1	1.1	0.42 (1.2 × 10^−7^)	95.4	1.2
4.1 (1.0 × 10^−6^)	98.7	10	4.0 (1.2 × 10^−6^)	96.4	12
10 (2.5 × 10^−6^)	98.0	25	10 (2.9 × 10^−6^)	96.4	29
100 (2.5 × 10^−5^)	96.1	248	100 (2.9 × 10^−5^)	96.4	290

## Data Availability

Data are contained within the article and Supplementary Material.

## Supplementary Materials

The following supporting information can be downloaded at: https://www.mdpi.com/article/10.3390/ph17010010/s1, Figure S1: Molar absorption coefficients (ε) of Cu-ATSM and Ni-ATSM were determined by using UV-Vis absorption spectra. (a): UV-Vis absorption spectra of Cu-ATSM, (b) UV-Vis absorption spectra of Ni-ATSM; Figure S2: Structure of DOTATATE; Figure S3: The structure of 1,10-dizaza-4,7-dithiodecane (log *K*_f_ value is 7.41 for Ni^2+^ and 10.70 for Cu^2+^).
