# Supplementary material for: Trace Metal Impurities Effects on the Formation of [64Cu]Cu-diacetyl-bis(N4-methylthiosemicarbazone) ([64Cu]Cu-ATSM)
Source: Pharmaceuticals (Basel). 2023 Dec 21;17(1):10. doi: 10.3390/ph17010010 (PMC10821298; doi:10.3390/ph17010010)
Supplement: Supplementary file 1 [file pharmaceuticals-17-00010-s001.zip › pharmaceuticals-2747457-supplementary.pdf]

pharmaceuticals

Supplementary Materials: Trace Metal Impurities Effects on the Formation of [^{64}Cu]Cu-diacetyl-bis(N^4-methylthiosemi-carbazone) ([^{64}Cu]Cu-ATSM)

Mitsuhiro Shinada [1,2,3,*], Hisashi Suzuki [2], Masayuki Hanyu [2], Chika Igarashi [2,3], Hiroki Matsumoto [2,3], Masashi Takahashi [1,2], Fukiko Hihara [2], Tomoko Tachibana [2,1], Chizuru Sogawa [2], Ming-Rong Zhang [2], Tatsuya Higashi [2], Hidemitsu Sato [3], Hiroaki Kurihara [3], Yukie Yoshii [2,3,*] and Yoshihiro Doi [1]

1 Faculty of Science, Toho University, Funabashi 274-8510, Japan; takahasi@chem.sci.toho-u.ac.jp (M.T.); yoshihiro.doi@sci.toho-u.ac.jp (Y.D.)
2 Institute for Quantum Medical Science, National Institutes for Quantum Science and Technology, Chiba 263-8555, Japan; suzuki.hisashi@qst.go.jp (H.S.); hanyu.masayuki@qst.go.jp (M.H.); cigaras2023@gmail.com (C.I.); matsumoto.hiroki2@qst.go.jp (H.M.); fukiko.hihara@gmail.com (F.H.); 7321201t@st.toho-u.jp (T.T.); sogawa.chizuru@qst.go.jp (C.S.); higashi.tatsuya@qst.go.jp (T.H.); zhang.ming-rong@qst.go.jp (M.-R.Z.)
3 Kanagawa Cancer Center, Kanagawa 241-8515, Japan; satohidemitsu@hotmail.com (H.S.); h-kurihara@kcch.jp (H.K.)
* Correspondence: 7123001s@st.toho-u.ac.jp (M.S.); yoshii.yukie@qst.go.jp (Y.Y.); Tel.: +81-43-206-3426

Citation: Shinada, M.; Suzuki, H.; Hanyu, M.; Igarashi, C.; Matsumoto, H.; Takahashi, M.; Hihara, F.; Tachibana, T.; Sogawa, C.; Zhang, M.-R.; et al. Tracer Metal Impurities Effects on the Formation of [^{64}Cu]Cu-diacetyl-bis (N^4-methylthiosemicarbazone) ([^{64}Cu]Cu-ATSM). *Pharmaceuticals* **2023**, *17*, 10. https://doi.org/10.3390/ph17010010

Academic Editor: Bianca Gutfilen

Received: 15 November 2023
Revised: 15 December 2023
Accepted: 19 December 2023
Published: 21 December 2023

Copyright: © 2023 by the authors. Licensee MDPI, Basel, Switzerland. This article is an open access article distributed under the terms and conditions of the Creative Commons Attribution (CC BY) license (https://creativecommons.org/licenses/by/4.0/).

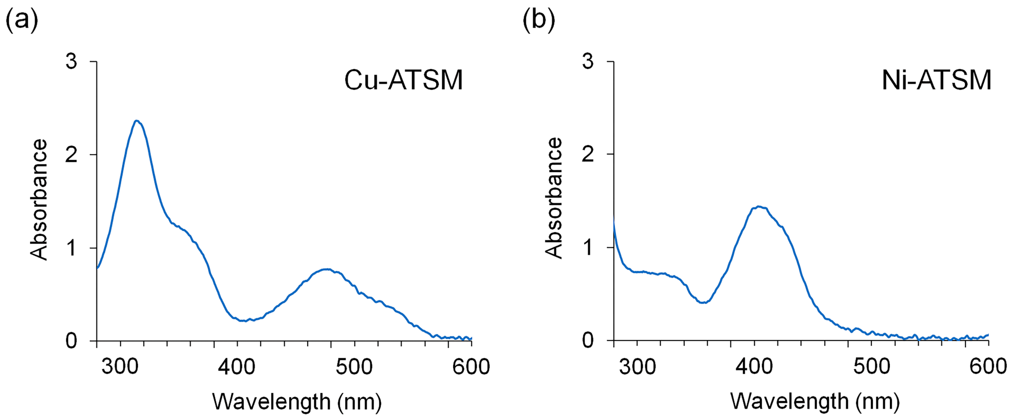

Figure S1. Molar absorption coefficients (ε) of Cu-ATSM and Ni-ATSM were determined by using UV-Vis absorption spectra. Concentrations of Cu- and Ni-ATSM were estimated using the molar extinction coefficients, ε, which were determined to be 7680 (Cu–ATSM at 477 nm) and 14300 cm M^{-1} (Ni-ATSM at 402 nm). (a): UV-Vis absorption spectra of Cu-ATSM, (b) UV-Vis absorption spectra of Ni-ATSM.

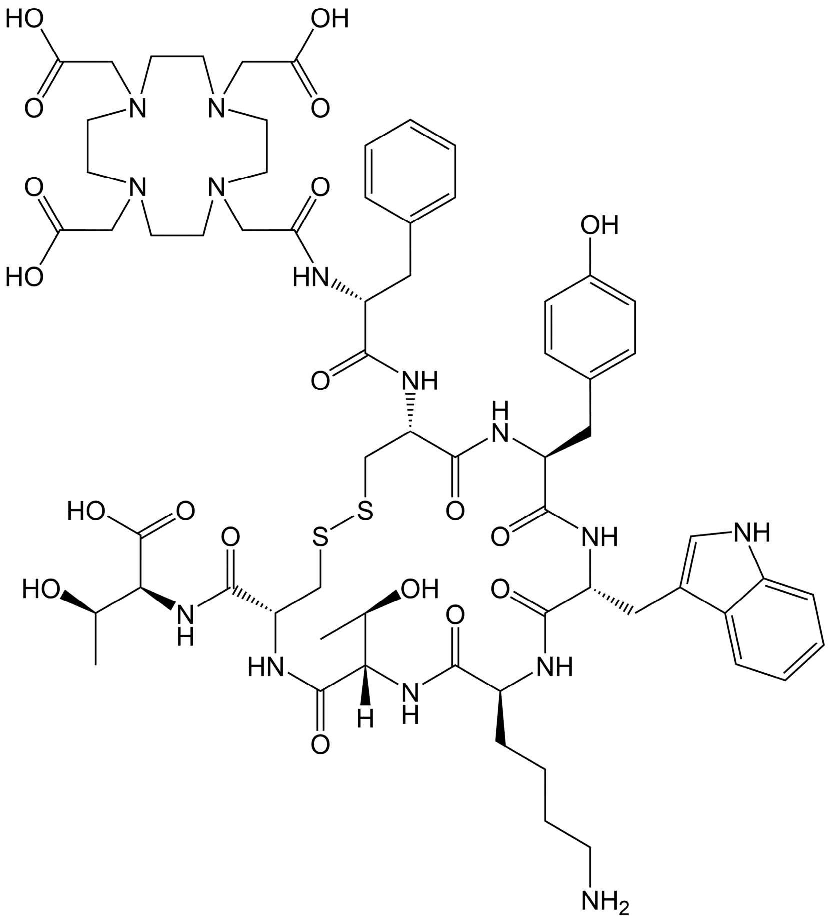

Oxodotreotide (DOTATATE)

Figure S2. Structure of DOTATATE.

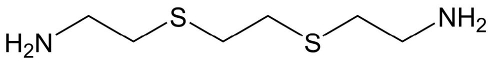

4,7-Dithia-1,10-diazadecane

Figure S3. The structure of 4,7-dithia-1,10-diazadecane (log K_f value is 7.41 for Ni^{2+} and 10.70 for Cu^{2+}) All authors have read and agreed to the published version of the manuscript.
